# Constipation symptoms are associated with worse cognitive outcomes in older adults without dementia

**DOI:** 10.3389/fnut.2025.1578181

**Published:** 2025-09-03

**Authors:** Xiaochang Liu, Juan Zhou, Xinyan Xie, Wenzhe Zheng, Dan Liu, Guirong Cheng, Feifei Hu, Junyi Wang, Cheng Cai, Jing Liu, Qianqian Nie, Shiyue Li, Dan Song, Yuyang Cui, Jingjing Zhang, Hua Meng, Wei Tan, Yan Zeng

**Affiliations:** ^1^Hubei Clinical Research Center for Alzheimer’s Disease, Tianyou Hospital Affiliated to Wuhan University of Science and Technology, Wuhan, China; ^2^Brain Science and Advanced Technology Institute, Wuhan University of Science and Technology, Wuhan, China; ^3^Department of Medical Imaging, Geriatric Hospital Affiliated to Wuhan University of Science and Technology, Wuhan, China; ^4^School of Public Health, Wuhan University of Science and Technology, Wuhan, Hubei, China

**Keywords:** constipation, mild cognitive impairment, constipation symptoms, cognitive domains, depressive symptoms

## Abstract

**Background:**

Constipation is correlated with cognitive impairment; however, the association of constipation symptoms with cognitive domains remains unclear. This study aimed to investigate this association.

**Methods:**

Participants aged 65 and older underwent neuropsychological, clinical, and laboratory examinations. Clinicians diagnosed constipation using the Rome IV criteria. Multivariate logistic regression assessed the odds ratios (ORs) and 95% confidence intervals (CIs) for mild cognitive impairment (MCI) and multi-domain cognitive impairments in relation to constipation and its specific symptoms. Mediation analysis was conducted to examine the effects of depressive symptoms.

**Results:**

Constipation was diagnosed in 9,417 participants without dementia [mean (standard deviation) age: 72.0 (5.6) years], while constipation symptoms were recorded in 3,344 individuals [mean (standard deviation) age: 72.6 (5.5) years]. Of the overall population, 1,895 (20.1%) were diagnosed with constipation. Constipation was associated with a higher MCI risk (OR: 1.177, 95% CI: 1.047–1.323), worse performance on language (OR: 1.133, 95% CI: 1.011–1.270), and executive function (OR: 1.386, 95% CI: 1.130–1.701). A higher MCI risk was associated with constipation symptoms: bowel movements every 3 or more days (OR: 1.391, 95% CI: 1.011–1.914), defecation difficulty (OR: 1.278, 95% CI: 1.002–1.629), and dry stools (OR: 1.326, 95% CI: 1.027–1.711). Prolonged bowel movements increased the risk of both memory and language impairment, but not MCI. Defecation difficulty was associated with memory impairment (OR: 1.309, 95% CI: 1.003–1.709), dry stools with language impairment (OR: 1.396, 95% CI: 1.088–1.791), and bowel movements every other day with executive impairment (OR: 1.761, 95% CI: 1.151–2.693). Depression mediated the association of constipation with global cognitive and language function.

**Conclusion:**

In the non-demented stage, constipation and its symptoms were associated with MCI and multi-domain cognitive impairments. These associations, along with depressive symptoms, should be further evaluated in large-scale population screenings to benefit cognitive impairment management.

## Introduction

With increasing incidence, dementia has become a leading cause of disability and death among older adults, posing a significant threat to their health and wellbeing worldwide (1–3). Due to the lack of effective treatments, early screening and interventions targeting modifiable risk factors and early clinical signs are paramount (1, 3). Constipation is a significant indication of gastrointestinal disorders that may involve malfunction in the autonomic nerve networks and is strongly associated with dementia (4–6), particularly Parkinson’s disease (PD) (7, 8). Constipation severity is associated with PD progression (9), mild cognitive impairment (MCI), poor performance on frontal executive function and visuospatial abilities (10), rapid progression of Alzheimer’s symptoms, and expansion of deep white matter lesions (11). Nevertheless, studies have mostly neglected specific constipation symptoms, such as defecation sensation, low bowel movement frequency, prolonged duration required for defecation, and hard and dry stool consistency. Furthermore, due to the uneven decline in cognitive domains during aging and neurodegeneration, assessing multiple cognitive domains, such as memory, language, attention, and executive function, can contribute to a comprehensive understanding of cognitive decline in older adults and facilitate the identification of individuals with specific cognitive domain impairments (12). However, the associations among constipation symptoms, specific cognitive subdomains, and potential mediating variables remain largely unknown.

Mild cognitive impairment (MCI) serves as the prodromal, intermediary phase bridging healthy aging and dementia, presenting a crucial “window of opportunity” that researchers and clinicians perceive as a potential point for intervention to hinder the progression toward dementia (13, 14). Targeting domain-specific cognitive changes associated with constipation symptoms may help refine this window, allowing for earlier and more personalized interventions. We hypothesized that older adults with constipation would have higher odds of MCI and multidomain cognitive impairment compared to those without constipation, and that specific constipation symptoms would be differentially associated with poorer performance in memory, language, and executive function domains. Geriatric depression is a critical risk factor for dementia and often coexists with gastrointestinal diseases (15, 16). An investigation utilizing data sourced from the UK Biobank has revealed a prospective correlation between constipation and an increased likelihood of developing depression (17). However, the indirect influence of depression on the associations of constipation with MCI and specific cognitive domains has not yet been confirmed. This study also aimed to investigate whether geriatric depression mediates the relationship between constipation and cognitive function. Clarifying the associations between specific constipation symptoms and distinct cognitive domains holds direct clinical relevance. If certain symptoms are linked to a specific cognitive domain, clinicians may prioritize these symptoms as red flags for proactive interventions. In addition, understanding such specificity could guide mechanistic research, as different cognitive domains map to distinct neural circuits that may interact with gut–brain pathways variably. This approach aligns with the growing emphasis on precision medicine in dementia prevention.

## Subjects and methods

### Study design and participants

**Figure 1:**
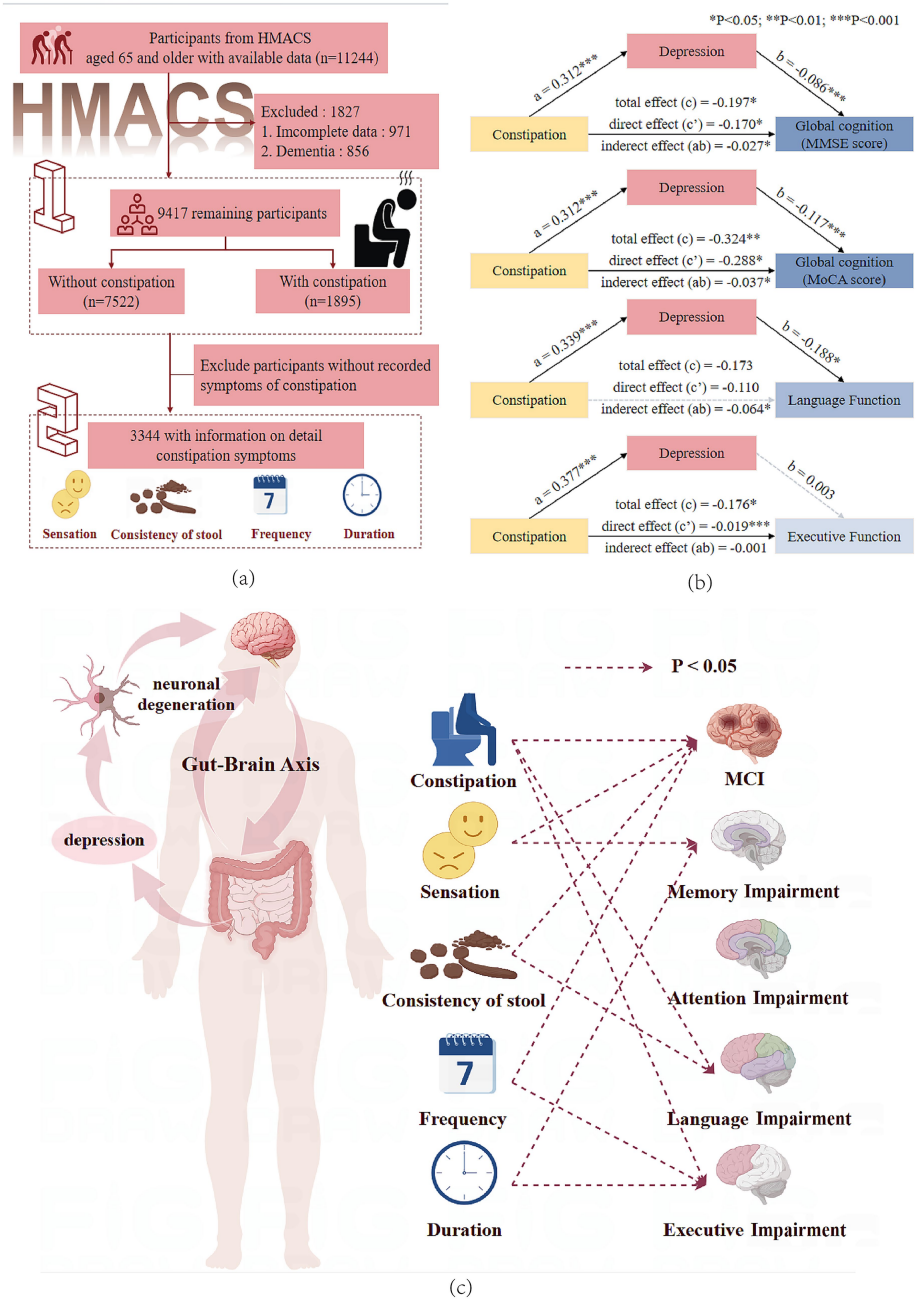
**(a)** Participant flowchart. **(b)** Mediation effect of depressive symptoms on the association between constipation and global cognitive, language, and executive function. MMSE, Mini-Mental State Examination; MoCA, Montreal Cognitive Assessment. *The statistical significance threshold was set at *p* < 0.05 (**p < 0.01, ***p < 0.001). **(c)** This figure shows the association between constipation symptoms and multi-domain cognitive impairments, supporting the existence of the gut–brain axis and the mediating role of depression.

We used data from the baseline survey (2018–2024) in the Hubei Memory and Aging Cohort Study (HMACS) (www.chictr.org.cn; registration number: ChiCTR1800019164) (18, 19). The HMACS is a large-scale population-based cohort study with more than 10,000 participants aged 65 or older from urban and rural villages in a less-developed area in China. Participants underwent detailed neuropsychological evaluations every 2 years, and their physical health was assessed during annual clinical examinations and laboratory tests. This study included 9,417 participants after excluding 1,827 due to incomplete constipation data or a diagnosis of dementia (including Parkinson’s disease). Specific constipation-type symptoms were documented for 3,344 individuals as data collection for these symptoms commenced after 2022 (Figure 1). This study was approved by the Medical Ethics Committee of the Wuhan University of Science and Technology (Protocol number: 201845). All the participants provided written informed consent.

### Constipation assessment

Constipation-type symptoms, including defecation frequency, duration, sensation, and stool consistency, were recorded by the researchers during face-to-face interviews. Constipation was diagnosed by clinicians based on the Rome IV criteria (Supplementary Table S1).

### Neuropsychological assessment

Participants underwent a detailed neuropsychological evaluation using a battery of tests, including two tests for global cognitive function (Mini-Mental State Examination [MMSE] and Basic Montreal Cognitive Assessment [MoCA-B]) (20) and four tests for specific cognitive domains: memory (Auditory Verbal Learning Test with immediate and long-delayed free recall) (21), language (semantic fluency) (Animal Fluency Test) (22), attention (Digit Span Forward and Digit Span Backward) (23), and executive functions (Trail Making Test A) (24). The executive function score resulted from a negative log transformation of the completion time of the Trail Making Test B. The language score is the number of correct answers, with higher scores representing better cognitive performance. The Activities of Daily Living Scale was used to evaluate the basic and instrumental functional abilities. A neurological expert panel evaluated all participants’ data and reached a consensus on the diagnoses according to the Diagnostic and Statistical Manual of Mental Disorders, IV criteria (25). MCI was diagnosed using the modified Petersen Criteria (14). Domain-specific impairments were assessed using standardized protocols and domain-specific screening scales, with specific criteria detailed in Supplementary Table S2.

### Covariates

A series of essential covariates associated with constipation and cognition were obtained from the baseline HMACS. Sociodemographic characteristics (sex, age, education, marital status, and residence location), lifestyle factors (smoking, alcohol consumption, vegetable intake, fruit intake, physical exercise, and intellectual activity), and physician-diagnosed clinical conditions (hypertension, diabetes, coronary heart disease, and hyperlipidemia) were confirmed by a review of electronic medical records and collected. The 15-item Geriatric Depression Scale was used as a depression screening instrument. Detailed definitions and information regarding the covariates are presented in Supplementary Table S3.

### Statistical analysis

SPSS Statistics 26 (IBM Corp., Armonk, NY, United States) and R 4.3.2 (R Development Core Team) were used for statistical analyses and graphing. Means and standard deviation (SD) were used to describe continuous variables, and numbers and percentages were used to describe categorical variables. The characteristics of the study participants in Table 1 were compared using the t-test for continuous variables and the chi-squared test for categorical variables. The associations among constipation, its specific symptoms, and cognitive impairments were investigated using multivariate logistic regression models adjusted for demographic factors, lifestyle habits, and medical history to control for potential confounding effects among the risk factor variables. The PROCESS procedure was used to assess whether depression mediated the association between constipation and cognitive function. The total, direct, and indirect effects were estimated using 5,000 bootstrap samples. To evaluate the robustness of the results, we conducted two sensitivity analyses: (1) incorporating age as a continuous variable into the model to counter partial information loss and (2) further controlling for the covariate body mass index (BMI) to reduce confounding effects. A *p*-value of <0.05 indicated statistical significance.

**Table 1:** Participant characteristics by constipation status.

Characteristics	Participants	Without constipation	Constipation	χ2/t	*p*-value
Overall	9,417 (100.0)	7,522 (79.9)	1,895 (20.1)		
Sex					
Male	4,373 (46.4)	3,567 (47.4)	806 (42.5)	14.539	<0.001
Female	5,044 (53.6)	3,955 (52.6)	1,089 (57.5)		
Age group (years)					
≤70	4,490 (47.7)	3,689 (49.0)	801 (42.3)	44.778	<0.001
71–80	4,046 (43.0)	3,192 (42.4)	854 (45.1)		
≥81	881 (9.4)	641 (8.5)	240 (12.7)		
Education (years)					
≤6	3,399 (36.1)	2,585 (34.4)	814 (43.0)	58.498	<0.001
6–12	4,587 (48.7)	3,721 (49.5)	866 (45.7)		
>12	1,431 (15.2)	1,216 (16.2)	215 (11.3)		
Marital status					
Married	7,185 (76.3)	5,809 (77.2)	1,376 (72.6)	26.996	<0.001
Unmarried	193 (2.0)	132 (1.8)	61 (3.2)		
Divorced/widowed	2,389 (24.9)	1,877 (24.1)	512 (28.7)		
Residence location					
Rural	3,101 (32.9)	2,330 (31.0)	771 (40.7)	64.620	<0.001
Urban	6,316 (67.1)	5,192 (69.0)	1,124 (59.3)		
Smoking	2,711 (28.8)	2,177 (28.9)	534 (28.2)	0.429	0.512
Alcohol consumption	2,603 (27.6)	2,108 (28.0)	495 (26.1)	2.741	0.098
Vegetable intake	9,269 (98.4)	7,420 (98.6)	1849 (97.6)	11.233	0.001
Fruit intake	5,933 (63.0)	4,848 (64.5)	1,085 (57.3)	33.617	<0.001
Physical exercise	6,328 (67.2)	5,206 (69.2)	1,122 (59.2)	68.696	<0.001
Intellectual activity	4,639 (49.3)	3,829 (50.9)	810 (42.7)	40.324	<0.001
Hypertension	4,883 (51.9)	3,878 (51.6)	1,005 (53.0)	1.326	0.250
Diabetes	1,648 (17.5)	1,256 (16.7)	392 (20.7)	16.677	<0.001
Coronary disease	1,580 (16.8)	1,168 (15.5)	412 (21.7)	41.854	<0.001
Hyperlipidemia	2,374 (25.2)	1,838 (24.4)	536 (28.3)	11.900	0.001
Depression	433 (4.6)	285 (3.8)	148 (7.8)	55.795	<0.001
MCI	2,752 (29.2)	2,077 (29.2)	675 (35.6)	46.927	<0.001
Mean (SD)					
Age (years)	72.00 (5.60)	71.78 (5.43)	72.90 (6.00)	−7.40	<0.001
Education (years)	8.08 (5.13)	8.30 (5.09)	7.19 (5.18)	8.320	<0.001
GDS	1.23 (1.62)	1.14 (1.49)	1.57 (1.99)	−8.79	<0.001
ADL	20.78 (1.90)	20.69 (1.70)	21.14 (2.52)	−7.33	<0.001
MMSE	26.67 (3.74)	26.84 (3.64)	26.02 (4.06)	8.06	<0.001
MoCA	23.16 (4.96)	23.40 (4.88)	22.19 (5.13)	9.28	<0.001

OR, odds ratio, CI, confidence interval.

## Results

### Characteristics of participants

A total of 9,417 participants without dementia [mean (SD) age: 72.0 (5.6) years] were included in the primary analysis. Of them, 1,895 (20.10%) self-reported constipation, 5,044 were women (53.6%), 3,101 resided in rural areas (32.9%), and 7,185 were married (76.3%). Participants with constipation were more likely to be female, older, with lower educational attainment, unmarried/divorced/widowed, and reside in rural areas (Table 1). Supplementary Tables S4, S5 present the main baseline characteristics of the population according to the MCI status. A total of 3,344 participants were included in the analysis to investigate the association between specific constipation symptoms and MCI as well as cognitive impairments across domains. A comparison of the baseline characteristics between subpopulations and the overall population is presented in Supplementary Table S6.

### Associations of constipation with MCI and cognitive domains

The results of the multivariate regression analysis are presented in Table 2. After adjusting for demographic factors (Model 2) and all covariates (Model 3) listed in Table 2, constipation was significantly associated with a higher risk of MCI (OR: 1.177, 95% CI: 1.047–1.323, *p* = 0.006), language impairment (OR: 1.133, 95% CI: 1.011–1.270, *p* = 0.031), and impaired executive function (OR: 1.386, 95% CI: 1.130–1.701, *p* = 0.002).

**Table 2:** Association between constipation and MCI and cognitive domains.

Characteristics	Model 1^a^		Model 2^b^		Model 3^c^	
	OR [95% CI]	*P*-value	OR [95% CI]	*P*-value	OR [95% CI]	*P*-value
MCI	1.450	<0.001	1.212	0.001	1.177	0.006
	[1.304, 1.614]		[1.080, 1.360]		[1.047, 1.323]	
Memory impairment	1.275	<0.001	1.149	0.059	1.125	0.116
	[1.116, 1.458]		[0.995, 1.328]		[0.971, 1.304]	
Executive impairment	1.514	<0.001	1.408	0.001	1.386	0.002
	[1.260, 1.820]		[1.151, 1.722]		[1.130, 1.701]	
Language impairment	1.221	<0.001	1.169	0.006	1.133	0.031
	[1.093, 1.364]		[1.045, 1.309]		[1.011, 1.270]	
Attention impairment	1.280	0.002	0.966	0.717	0.955	0.630
	[1.095, 1.498]		[0.803, 1.163]		[0.791, 1.153]	

OR, odds ratio, CI, confidence interval.

a Crude model.

b Adjusted variables: sex, age, education, residence, and marital status.

c Adjusted variables: sex, age, education, residence, marital status, smoking, alcohol consumption, vegetable intake, fruit intake, physical exercise, intellectual activity, depression, hypertension, diabetes, coronary heart disease, and hyperlipidemia.

### Associations of specific constipation symptoms with MCI and cognitive domains

**Figure 2:**
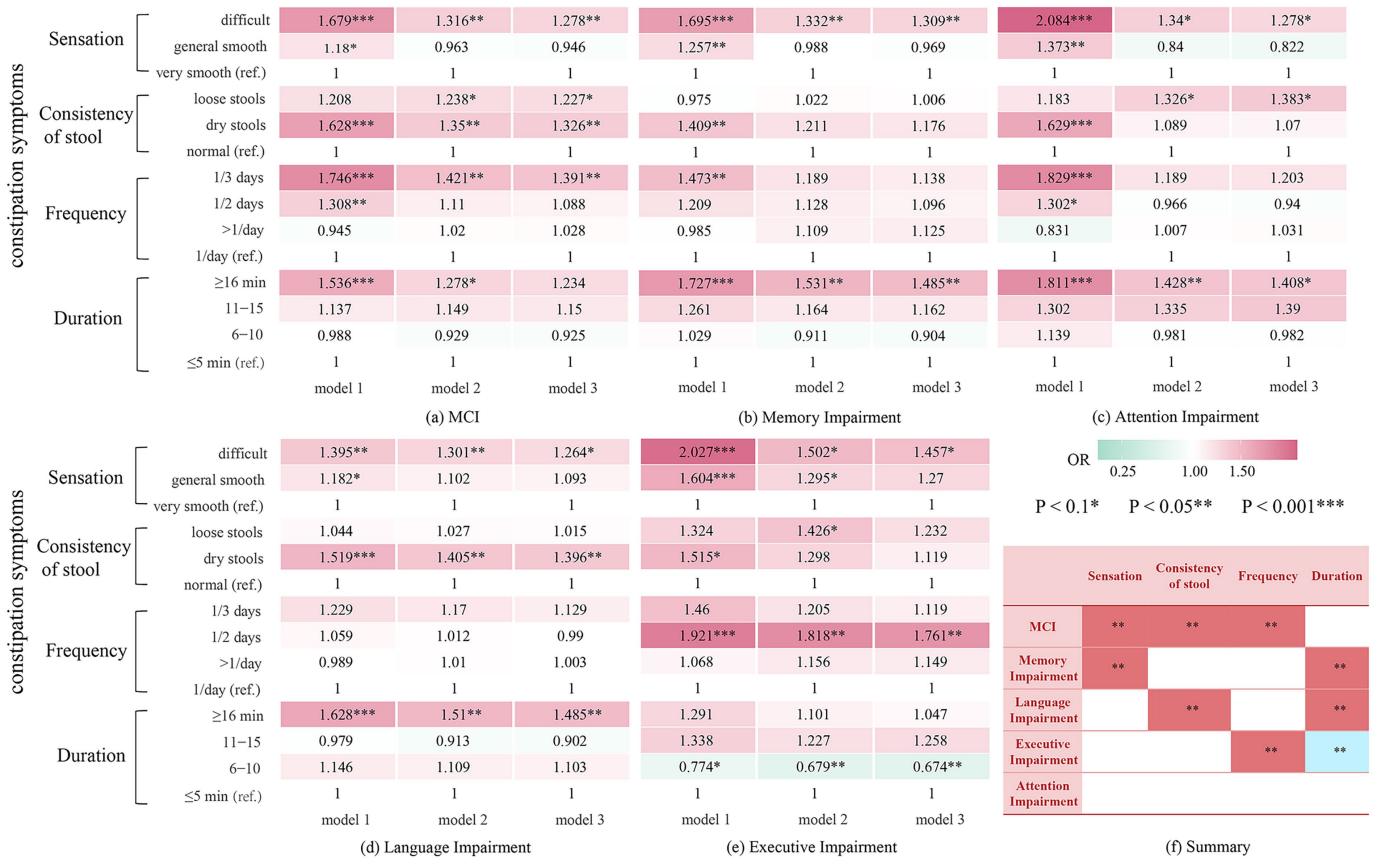
Results of stepwise adjusted logistic regression analysis on the correlation between constipation and MCI and impairment among cognitive domains. **(a)** Relationship between constipation symptoms and MCI. **(b)** Relationship between constipation symptoms and memory impairment. **(c)** Relationship between constipation symptoms and attention impairment. **(d)** Relationship between constipation symptoms and language impairment. **(e)** Relationship between constipation symptoms and executive impairment. **(f)** A summary of **(a–e)**. MCI, Mild Cognitive Impairment. Model 1: crude model; Model 2: Adjusted for sex, age, education, residence, and marital status Model 3: Adjusted for sex, age, education, residence, marital status, smoking, alcohol consumption, vegetable intake, fruit intake, physical exercise, intellectual activity, depression, hypertension, diabetes, coronary heart disease, and hyperlipidemia.

A total of 3,344 participants were included in the analysis to investigate the associations of specific constipation symptoms with MCI and cognitive impairment across domains. In the fully adjusted model, having bowel movements once every 3 days or longer (OR: 1.391, 95% CI: 1.011–1.914, *p* = 0.043), experiencing difficulty in defecation (OR: 1.278, 95% CI: 1.002–1.629, *p* = 0.048), and experiencing dry stool (OR: 1.326, 95% CI: 1.027–1.711, *p* = 0.030) were closely associated with MCI (Table 3). Although prolonged bowel movement was not significantly associated with MCI, it increased the risk of memory (OR: 1.485, 95% CI: 1.097–2.009, *p* = 0.010) and language impairment (OR: 1.485, 95% CI: 1.137–1.939, *p* = 0.004) (Supplementary Table S7 and Figure 2). Defecation experience, stool consistency, and bowel movement frequency were significantly associated with corresponding cognitive impairments across domains: defecation difficulty was associated with worse performance on memory (OR: 1.309, 95% CI: 1.003–1.709, *p* = 0.047), passing dry stool was associated with language impairment (OR: 1.396, 95% CI: 1.088–1.791, *p* = 0.009), and bowel movements every other day was associated with executive impairment (OR: 1.761, 95% CI: 1.151–2.693, *p* = 0.009). The association between constipation symptoms and some cognitive domain-specific impairments approached significance, with *p* < 0.1, indicated by an asterisk (*) in Figure 2.

**Table 3:** Association between constipation symptoms and MCI.

Characteristics		Model 1^a^		Model 2^b^		Model 3^c^	
		OR [95% CI]	*P*-value	OR [95% CI]	*P*-value	OR [95% CI]	*P*-value
Duration	≤5 min	1 [Reference]		1 [Reference]		1 [Reference]	
	6–10	0.988 [0.831, 1.175]	0.894	0.929 [0.773, 1.117]	0.759	0.925 [0.768, 1.113]	0.408
	11–15	1.137 [0.843, 1.533]	0.540	1.149 [0.836, 1.578]	0.392	1.150 [0.834, 1.586]	0.393
	≥16 min	1.536 [1.192, 1.978]	0.001	1.278 [0.974, 1.677]	0.077	1.234 [0.937, 1.625]	0.134
Frequency	1/day	1 [Reference]		1 [Reference]		1 [Reference]	
	>1/day	0.945 [0.785, 1.137]	0.546	1.020 [0.839, 1.241]	0.842	1.028 [0.844, 1.253]	0.783
	1/2 days	1.308 [1.026, 1.667]	0.030	1.110 [0.856, 1.439]	0.429	1.088 [0.838, 1.414]	0.526
	1/>3 days	1.746 [1.300, 2.346]	<0.001	1.421 [1.037, 1.947]	0.029	1.391 [1.011, 1.914]	0.043
Stool consistency	Normal	1 [Reference]		1 [Reference]		1 [Reference]	
	Dry stools	1.628 [1.284, 2.062]	<0.001	1.350 [1.048, 1.739]	0.020	1.326 [1.027, 1.711]	0.030
	Loose stools	1.208 [0.965, 1.514]	0.100	1.238 [0.975, 1.572]	0.079	1.227 [0.964, 1.561]	0.096
Sensation	Very smooth	1 [Reference]		1 [Reference]		1 [Reference]	
	General smooth	1.180 [0.995, 1.400]	0.057	0.963 [0.802, 1.156]	0.685	0.946 [0.786, 1.138]	0.554
	Difficult	1.679 [1.343, 2.098]	<0.001	1.316 [1.037, 1.670]	0.024	1.278 [1.002, 1.629]	0.048

OR, odds ratio, CI, confidence interval.

a Crude model.

b Adjusted variables: sex, age, education, residence, and marital status.

c Adjusted variables: sex, age, education, residence, marital status, smoking, alcohol consumption, vegetable intake, fruit intake, physical exercise, intellectual activity, depression, hypertension, diabetes, coronary heart disease, and hyperlipidemia.

### The mediating effect of depressive symptoms on the relationship between constipation and cognitive performance

PROCESS Model 4 was used to test for the indirect influence of depressive symptoms on the relationship between constipation and global cognitive function, semantic fluency, or executive function. Depression had 13.7 and 11.3% mediating effects on the impact of constipation on lower MMSE and MoCA scores, respectively (Figure 1b, Supplementary Table S8). Moreover, depression mediated the association between constipation and low semantic fluency scores (mediated proportion: 36.6%) but had no mediating effect on the relationship between constipation and low executive function scores.

### Sensitivity analyses

The results of the sensitivity analyses are presented in the Supplementary materials. First, age was included as a continuous variable (Supplementary Tables S9, S10), and the main findings remained consistent. Subsequently, we further included a covariate to control for confounding factors. The associations of constipation with MCI and domain-specific cognitive impairment remained stable (Supplementary Table S11). Notably, the association between bowel movements occurring once every 3 days or longer and defecation difficulty approached but did not reach significance in the MCI group (Supplementary Table S12).

## Discussion

### Main findings

This large-scale community-based study on older adults confirmed that constipation or its symptoms exhibited complex associations with worse cognitive outcomes as early as the non-demented stage in older adults. Importantly, even after adjusting for potential confounders, including diet, physical activity, and cardiometabolic conditions, constipation remained closely associated with higher MCI risk and worse performance in language and executive function. This suggests that constipation may serve as a unique health indicator in MCI, extending beyond a mere reflection of general health behaviors or comorbidities. Specifically, bowel movements every 3 or more days, defecation difficulty, and passing dry stools were associated with higher MCI risk. Various constipation symptoms, including defecation duration, were associated with worse performance on single or multiple cognitive domains. Geriatric depression mediated the association of constipation and worse performance on global cognitive function and semantic fluency.

Dysfunction of the gut–brain axis is a crucial factor in neurological diseases (26–28). Constipation in the older population may be accompanied by alterations in the gut microbiota, which might mechanistically contribute to neuroinflammation, insulin resistance (29, 30), and subsequent neuropathogenesis (31). Consistent with previous findings (7, 10, 32), this study revealed an association between constipation and MCI. An investigation of 427 individuals diagnosed with PD revealed a correlation between constipation and MCI, impaired executive function, and visual–spatial impairment (10). Although our study excluded individuals diagnosed with PD, a similar association between constipation and executive function was observed in the population without dementia, suggesting that this association does not exist only in PD patients but also in all-cause MCI patients. Unlike previous studies, this study is the first to reveal a correlation between constipation and worse performance on semantic fluency and executive functions. Studies have shown that chronic constipation disrupts gut microbiota balance and elevates serum inflammatory factors. These cytokines may cross the blood–brain barrier, activate prefrontal microglia, and downregulate synaptophysin expression, potentially impairing executive function (33). Chronic constipation is often accompanied by anxiety and depression (17), which may further affect executive function by altering neurotransmitter release and neural pathway signaling. The frontal lobe, rich in dopamine-sensitive neurons, is critical for executive function, and its atrophy or dysfunction in constipation patients may lead to cognitive decline (34). Additionally, the vagus nerve, as a core pathway of the gut–brain axis, its dysfunction may affect the prefrontal–striatal circuit. Vagus nerve stimulation has been shown to enhance prefrontal activity, improving executive and language functions, particularly in temporal processing and cognitive flexibility (35, 36).

This study is the first attempt to offer a more elaborate and thorough categorization of various constipation symptoms, including defecation sensation, frequency, duration, and stool consistency, and to demonstrate their complex epidemiological associations with the cognitive domain. In a cross-sectional study by Huang et al. involving 751 community-residing individuals in Singapore, participants who reported bowel movements four times per week or more demonstrated a lower risk of MCI, with an odds ratio (OR) of 0.58 (95% CI: 0.36–0.94), in comparison to those having bowel movements three times per week or less (37). In three large U.S. cohort studies involving older adults with a mean baseline age of 67.2 years, Ma et al. found that lower bowel movement frequency was associated with worse cognition (38). The conclusions of the present study are consistent with these findings. Research has found that differences in bowel movement frequency are closely associated with specific changes in gut microbiota composition (38). In individuals with lower bowel movement frequency, the abundance of bacteria with anti-inflammatory and metabolic regulatory functions is significantly reduced. These bacteria typically participate in the synthesis of short-chain fatty acids such as butyrate, which play a crucial role in maintaining the integrity of the blood–brain barrier and inhibiting neuroinflammation (39). Dysbiosis of the gut microbiota may exacerbate oxidative stress and neuroinflammation by reducing the production of neuroprotective metabolites, thereby damaging the prefrontal–striatal loop associated with executive function.

This study is the first to reveal the association between hard stools and MCI and language impairment, as well as the association between difficulty in defecation and memory impairment. Stool consistency is strongly associated with the richness, composition, enterotypes, and bacterial growth rates (40) of the gut microbiota. Dietary factors and chronic stress may contribute to hard stools (41), as prolonged colonic transit time leads to increased water absorption, resulting in harder stools. Individuals with hard stools have a doubled all-cause mortality risk compared to those with normal stools (HR = 2.00, 95%CI:1.48–2.70) (42). Notably, no direct evidence previously linked stool consistency or defecation sensation to cognitive impairment. Feeling difficulty in defecation may trigger recurrent physical discomfort and psychological stress, activating the hypothalamic–pituitary–adrenal axis and elevating cortisol levels (43). Chronic stress not only exacerbates systemic inflammation but also inhibits hippocampal neurogenesis, disrupts synaptic plasticity, and leads to memory impairment (44). Furthermore, we discovered for the first time that prolonged defecation duration (>15 min) and defecation difficulty were associated with MCI and memory and language impairment, suggesting that health screening among the aging population should also focus on the emergence of specific constipation symptoms rather than merely the diagnosis of constipation.

We also elucidated the mediating role of depression in the association between constipation and worse performance in global cognitive function as well as language impairment. Based on the gut–brain axis theory, intestinal diseases may lead to dementia by affecting the central nervous system. The vagus nerve, one of the largest nerves connecting the gastrointestinal and nervous systems, is closely associated with psychological stress and depression (7, 45). We hypothesized that constipation directly impacts MCI and indirectly leads to MCI by triggering or exacerbating depressive symptoms. However, our results indicate that the association between constipation and language impairment was mediated by depression. This mediation may be related to the increased sensitivity of language function to emotional states. Language function is a component of cognitive function susceptible to emotional disturbances. Studies have shown that depression and Alzheimer’s disease share common patterns and neurobiological bases for language dysfunction, and that intensive language repair training targeting shared neural networks is beneficial for patients with both depression and Alzheimer’s disease (46). These findings suggest that in clinical practice, clinicians treating older patients with constipation should monitor their emotional status and cognitive function and promptly identify and intervene in depressive symptoms.

### Strengths and limitations

The advantage of this study is that it is the latest large-scale epidemiological study on specific constipation symptoms and multi-domain cognitive impairments in older Chinese people without dementia. Furthermore, we explored the mediating role of depression, which provided new insights into the relationship between constipation and cognitive function. This study has limitations. First, due to the nature of the cross-sectional study, we could not discern the exact age of onset and temporal changes in constipation and cognitive impairment, making it impossible to establish a causal relationship between constipation, its specific symptoms, and cognitive impairment. Second, there may be some unknown confounding variables in the association between constipation and cognitive impairment, and residual confounding might still exist.

## Conclusion

In summary, even in the non-demented stage, constipation and specific constipation symptoms showed complex associations with MCI and multi-domain cognitive impairments. Depression may partially explain the mechanistic association between constipation and cognitive function. These findings underscore the significance of evaluating multi-domain symptoms of constipation and cognition, along with depression symptoms, in large-scale population screenings. Further studies should consider additional gut-related variables, such as dietary patterns, gut microbiota composition, and inflammatory levels, to modulate the relationship between constipation and cognitive function. Longitudinal studies are necessary to validate the causal relationship between constipation and cognitive decline in older adults. The findings in this study could provide insight into formulating new approaches to improve cognitive impairment management.

## Data Availability

The raw data supporting the conclusions of this article will be made available by the authors, without undue reservation.
