# Supplementary material for: Constipation symptoms are associated with worse cognitive outcomes in older adults without dementia
Source: Front Nutr. 2025 Sep 3;12:1578181. doi: 10.3389/fnut.2025.1578181 (PMC12442423; doi:10.3389/fnut.2025.1578181)
Supplement: Supplementary file 1 [file Table_1.DOC]

| **Table S1. Definition of Constipation and its Symptoms.** | |
| --- | --- |
| Characteristics | Method |
| Constipation | Clinical diagnosis was made by clinicians based on the patient's self-reported symptoms, such as reduced stool volume, hard stool consistency, difficulty in defecation, and less than three bowel movements per week. |
| Duration | Participants were asked about the average duration of each bowel movement.(Record the specific minutes) 1=≤5min 2=6-10min 3=11-15min 4=≥16min |
| Frequency | Participants were asked about the specific frequency of bowel movements. 1=1/day 2=>1/day 3=1/2 days 4=1/>3 days |
| Consistency of stool | Participants were asked to identify and select the category that best describes their stool consistency over the past week using the Bristol Stool Form Scale.The Bristol Stool Form Scale comprises seven categories ranging from 1 (separate hard lumps, like nuts) to 7 (watery, no solid pieces). 1=normal (Bristol 3-5) 2=dry stools (Bristol 1-2) 3=loose stools (Bristol 6-7) |
| Sensation | Participants were asked about their feelings during bowel movements. 1=very smooth 2=general smooth 3=difficult |

**Table S2.** The assessment of four domain-specific cognitive functions

| Cognitive domains | Assessment tools | Domain threshold |
| --- | --- | --- |
| Memory | Auditory Verbal Learning Test with long-delayed free recall | Delayed recall scores are used to evaluate memory. Thresholds are determined by subtracting 1.0 standard deviation (SD) from the mean score within each age group: <70 years, 70–79 years, and ≥80 years.   1. If aged 60-69 years old, a score of ≤ 3 indicates impairment. 2. If aged 70-79 years old, a score of ≤ 2 indicates impairment. 3. If aged 80 years old and above, a score of ≤ 1 indicates impairment. |
| Attention | Digit Span Test | The total score on the forward and backward conditions of the digit span test less than 9 indicates impairment. |
| Executive function | Trail Making Test A | In the Trail Making Test A, the test results are classified as abnormal under the following time thresholds based on different educational attainment levels and age groups:   1. For individuals with an educational attainment of ≤ 9 years:   If aged 60 - 69 years old, a time > 90 seconds indicates impairment.  If aged 70 years old and above, a time > 120 seconds indicates impairment.   1. For individuals with an educational attainment of ≥ 10 years:   If aged 60 - 69 years old, a time > 80 seconds indicates impairment.  If aged 70 years old and above, a time > 85 seconds indicates impairment. |
| Language | Animal Fluency Test | In the Animal Fluency Test, the result is considered abnormal according to the following thresholds based on different educational attainment levels:   1. For those who are illiterate, a score of ≤ 8 indicates impairment. 2. For individuals with a primary school education, a score of ≤ 10 indicates impairment. 3. For those with a junior high school education, a score of ≤ 12 indicates impairment. 4. For individuals with a senior high school education, a score of ≤ 13 indicates impairment. 5. For those with a university education, a score of ≤ 14 indicates impairment. |

| **Table S3. Definition of Covariates.** | |
| --- | --- |
| **Characteristics** | **Method** |
| sex | Based on personal ID information |
| age | Comprehensive judgment based on personal ID information and self-reported information |
| education | Self-reported |
| residence | Comprehensive judgment based on current residential area and self-reported information |
| marital status | Self-reported |
| Body Mass Index (BMI) | The participants' height and weight were measured, and it is represented as BMI = weight (kg) / [height (m)]². |
| smoking | Self-reported question on smoking history. |
| drinking | Self-reported question on drinking history. |
| eating vegetables | Participants were asked to rate the frequency of engagement in eating vegetables. Having vegetable intake for at least 3 days per week is considered as eating vegetables regularly |
| eating fruits | Participants were asked to rate the frequency of engagement in eating vegetables. Having fruits intake for at least 3 days per week is considered as eating fruits regularly |
| physical exercise | Participants were asked to rate the frequency of engagement in each of five activities (e.g., walking for exercise, jogging, dancing, biking, and ball games) during the past 2 weeks on a five-point scale, with 4 indicating participation in the activity every day or approximately every day, and 0 indicating participation once a month or less, along with recording the mean time per occasion. The minutes in each activity were then summed and divided by 120 to yield a summary measure of hours per week of physical activity. Physical exercise was defined as engaging in at least 90 minutes of moderate to vigorous activity per week. |
| intellectual activity | Participants were asked to rate the frequency of engaging in cognitively stimulating activities (reading books, reading newspapers, playing chess, playing cards, playing games, handwriting, etc.) on a five-point scale, with 4 indicating participation in the activity every day or approximately every day, and 0 indicating participation once a month or less. Intellectual activity was defined as engaging in cognitively stimulating activity at least 3 times per week. |
| depression | We assessed depressive symptoms using a Chinese version Geriatric Depression Scale -15 in the past 2 weeks as previously described. Persons were asked if they had experienced each of 15 symptoms during this time perod and the score was the total number of items. Persons with 0–4 scores were defined as normal, 5–8 scores as mild depression, 8–11 points as moderate depression, and 12–15 as severe depression (5 scores or above is considered as possible depression). |
| hypertension | Preferred diagnosis of hypertension relied on self-reported clinical diagnosis or the use of any anti-hypertensive medication within two weeks. If either was reported, hypertension was defined by measuring the value of blood pressure (systolic BP ≥140 mmHg and/or diastolic BP ≥ 90 mmHg) based on the National Guidelines for the Prevention and Management of Hypertension at Basic Level 2020. |
| diabetes | The preferred was to use self-reported clinical diagnosis of diabetes; if self-reported data were not available, fasting blood glucose level (≥7) was used for definition. |
| coronary heart disease | Self-reported diagnosis of coronary heart disease in later life and/or the use of any medication for treating coronary heart disease within 2 weeks. |
| hyperlipidemia | Self-reported diagnosis of hyperlipidemia in later life. |

| Table S4 Characteristics of the participants by MCI. (n=9417) | | | | | |
| --- | --- | --- | --- | --- | --- |
| Characteristics | Participants | cognitively normal | MCI | χ2/t | p value |
| Overall | 9417 (100.0) | 6665 (70.8) | 2752 (29.2) |  |  |
| Gender |  |  |  |  |  |
| Male | 4373 (46.4) | 3277 (49.2) | 1096 (39.8) | 68.338 | <0.001 |
| Female | 5044 (53.6) | 3388 (50.8) | 1656 (60.2) |
| Age group (years) |  |  |  |  |  |
| ≤70 | 4490 (47.7) | 3551 (53.3) | 939 (34.1) | 353.144 | <0.001 |
| 71-80 | 4046 (43.0) | 2657 (39.9) | 1389 (50.5) |
| ≥81 | 881 (9.4) | 457 (6.9) | 424 (15.4) |
| Education (years) |  |  |  |  |  |
| ≤6 | 3399 (36.1) | 1783 (26.8) | 1616 (58.7) | 872.867 | <0.001 |
| 6-12 | 4587 (48.7) | 3674 (55.1) | 913 (33.2) |
| ＞12 | 1431 (15.2) | 1208 (18.1) | 223 (8.1) |
| Marital status |  |  |  |  |  |
| Married | 7185 (76.3) | 5408 (81.1) | 1777 (64.6) | 296.063 | <0.001 |
| Unmarried | 193 (2.0) | 105 (1.6) | 88 (3.2) |
| Divorced/Widowed | 2389 (24.9) | 1152 (17.3) | 887 (32.2) |
| Residence location |  |  |  |  |  |
| Rural | 3101 (32.9) | 1716 (25.7) | 1385 (50.3) | 532.847 | <0.001 |
| Urban | 6316 (67.1) | 4949 (74.3) | 1367 (49.7) |
| Smoking | 2711 (28.8) | 2025 (30.4) | 686 (24.9) | 28.275 | <0.001 |
| Drinking | 2603 (27.6) | 1883 (28.3) | 720 (26.2) | 4.251 | 0.039 |
| Eating vegetables | 9269 (98.4) | 6562 (98.5) | 2707 (98.4) | 0.102 | 0.750 |
| Eating fruits | 5933 (63.0) | 4519 (67.8) | 1414 (51.4) | 225.328 | <0.001 |
| Physical exercise | 6328 (67.2) | 4757 (71.4) | 1571 (57.1) | 180.370 | <0.001 |
| Intellectual activity | 4639 (49.3) | 3751 (56.3) | 888 (32.3) | 449.297 | <0.001 |
| Hypertension | 4883 (51.9) | 3369 (50.5) | 1514 (55.0) | 15.567 | <0.001 |
| Diabetes | 1648 (17.5) | 1131 (17.0) | 517 (18.8) | 4.454 | 0.035 |
| Coronary disease | 1580 (16.8) | 1094 (16.4) | 486 (17.7) | 2.165 | 0.141 |
| Hyperlipidemia | 2374 (25.2) | 1769 (26.5) | 605 (22.0) | 21.459 | <0.001 |
| Depression | 433 (4.6) | 235 (3.5) | 198 (7.2) | 59.768 | <0.001 |

| Table S5 Characteristics of the participants by MCI in **subpopulation**. (n=3344) | | | | | |
| --- | --- | --- | --- | --- | --- |
| Characteristics | Participants | cognitively normal | MCI | χ2/t | p value |
| Overall | 3344 (100.0) | 2213 (66.2) | 1131 (33.8) |  |  |
| Gender |  |  |  |  |  |
| Male | 1499 (44.8) | 1095 (49.5) | 404 (35.7) | 57.297 | <0.001 |
| Female | 1845 (55.2) | 1118 (50.5) | 727 (64.3) |
| Age group (years) |  |  |  |  |  |
| ≤70 | 1393 (41.7) | 1020 (46.1) | 373 (33.0) | 67.550 | <0.001 |
| 71-80 | 1619 (48.4) | 1020 (46.1) | 599 (53.0) |
| ≥81 | 332 (9.9) | 173 (7.8) | 159 (14.1) |
| Education (years) |  |  |  |  |  |
| ≤6 | 1507 (45.1) | 783 (35.4) | 724 (64.0) | 262.144 | <0.001 |
| 6-12 | 1463 (43.8) | 1108 (50.1) | 355 (31.4) |
| ＞12 | 374 (11.2) | 322 (14.6) | 52 (4.6) |
| Marital status |  |  |  |  |  |
| Married | 2432 (72.7) | 1726 (78.0) | 706 (62.4) | 92.152 | <0.001 |
| Unmarried | 80 (2.4) | 46 (2.1) | 34 (3.0) |
| Divorced/Widowed | 832 (24.9) | 441 (19.9) | 391 (34.6) |
| Residence location |  |  |  |  |  |
| Rural | 1607 (48.1) | 934 (42.2) | 673 (59.5) | 89.737 | <0.001 |
| Urban | 1737 (51.9) | 1279 (57.8) | 458 (40.5) |
| Smoking | 943 (28.2) | 676 (30.5) | 267 (23.6) | 17.801 | <0.001 |
| Drinking | 941 (28.1) | 673 (30.4) | 268 (23.7) | 16.692 | <0.001 |
| Eating vegetables | 3287 (98.3) | 2179 (98.5) | 1108 (98.0) | 1.104 | 0.293 |
| Eating fruits | 1920 (57.4) | 1368 (61.8) | 552 (48.8) | 51.816 | <0.001 |
| Physical exercise | 2135 (63.8) | 1471 (66.5) | 664 (58.7) | 19.535 | <0.001 |
| Intellectual activity | 1208 (36.1) | 937 (42.3) | 271 (24.0) | 109.576 | <0.001 |
| Hypertension | 1814 (54.2) | 1197 (54.1) | 617 (54.6) | 0.065 | 0.799 |
| Diabetes | 626 (18.7) | 410 (18.5) | 216 (19.1) | 0.161 | 0.689 |
| Coronary disease | 618 (18.5) | 416 (18.8) | 202 (17.9) | 0.437 | 0.509 |
| Hyperlipidemia | 864 (25.8) | 591 (26.7) | 273 (24.1) | 2.576 | 0.109 |
| Depression | 190 (5.7) | 97 (4.4) | 93 (8.2) | 20.591 | <0.001 |

| **Table S6. Comparison of baseline characteristics between the total population and the subpopulation with reported constipation symptoms.** | | | | |
| --- | --- | --- | --- | --- |
| Characteristics | Total population | Subpopulation | χ2/t | p value |
| Overall | 9417 (100.0) | 3344 (100.0) |  |  |
| Gender |  |  |  |  |
| Male | 4373 (46.4) | 1499 (44.8) | 2.577 | 0.108 |
| Female | 5044 (53.6) | 1845 (55.2) |
| Age group (years) |  |  |  |  |
| ≤70 | 4490 (47.7) | 1393 (41.7) | 36.782 | <0.001 |
| 71-80 | 4046 (43.0) | 1619 (48.4) |
| ≥81 | 881 (9.4) | 332 (9.9) |  |  |
| Education (years) |  |  |  |  |
| ≤6 | 3399 (36.1) | 1507 (45.1) | 92.545 | <0.001 |
| 6-12 | 4587 (48.7) | 1463 (43.8) |
| ＞12 | 1431 (15.2) | 374 (11.2) |
| Marital status |  |  |  |  |
| Married | 7185 (76.3) | 2432 (72.7) | 16.961 | <0.001 |
| Unmarried | 193 (2.0) | 80 (2.4) |
| Divorced/Widowed | 2389 (24.9) | 832 (24.9) |
| Residence location |  |  |  |  |
| Rural | 3101 (32.9) | 1607 (48.1) | 242.516 | <0.001 |
| Urban | 6316 (67.1) | 1737 (51.9) |
| Smoking | 2711 (28.8) | 943 (28.2) | 0.418 | 0.518 |
| Drinking | 2603 (27.6) | 941 (28.1) | 0.306 | 0.580 |
| Eating vegetables | 9269 (98.4) | 3287 (98.3) | 0.276 | 0.599 |
| Eating fruits | 5933 (63.0) | 1920 (57.4) | 32.543 | <0.001 |
| Physical exercise | 6328 (67.2) | 2135 (63.8) | 12.413 | <0.001 |
| Intellectual activity | 4639 (49.3) | 1208 (36.1) | 171.566 | <0.001 |
| Hypertension | 4883 (51.9) | 1814 (54.2) | 5.668 | 0.017 |
| Diabetes | 1648 (17.5) | 626 (18.7) | 2.507 | 0.113 |
| Coronary disease | 1580 (16.8) | 618 (18.5) | 5.018 | 0.025 |
| Hyperlipidemia | 2374 (25.2) | 864 (25.8) | 0.513 | 0.474 |
| Depression | 433 (4.6) | 190 (5.7) | 6.241 | 0.012 |

| **Table S7. Association between constipation symptoms and cognitive domains. (n=3344)** | | | | | | | |
| --- | --- | --- | --- | --- | --- | --- | --- |
| **Characteristics** | | **Model 1**^a^ | | **Model 2**^b^ | | **Model 3**^c^ | |
|  |  | **OR  [95% CI]** | **P value** | **OR  [95% CI]** | **P value** | **OR  [95% CI]** | **P value** |
| **Outcome: Memory impairment** | | | | | | | |
| Duration | ≤5 min | 1 [Reference] |  | 1 [Reference] |  | 1 [Reference] |  |
|  | 6-10 | 1.029 [0.863, 1.227] | 0.752 | 0.911 [0.754, 1.102] | 0.339 | 0.904 [0.746, 1.096] | 0.408 |
|  | 11-15 | 1.261 [0.934, 1.702] | 0.130 | 1.164 [0.842, 1.611] | 0.358 | 1.162 [0.836, 1.615] | 0.393 |
|  | ≥16 min | 1.727 [1.309, 2.277] | <0.001 | 1.531 [1.137, 2.063] | 0.005 | 1.485 [1.097, 2.009] | 0.010 |
| Frequency | 1/day | 1 [Reference] |  | 1 [Reference] |  | 1 [Reference] |  |
|  | >1/day | 0.985 [0.816, 1.188] | 0.874 | 1.109 [0.906, 1.357] | 0.315 | 1.125 [0.917, 1.381] | 0.260 |
|  | 1/2 days | 1.209 [0.932, 1.568] | 0.153 | 1.128 [0.854, 1.491] | 0.397 | 1.096 [0.827, 1.453] | 0.523 |
|  | 1/3 days | 1.473 [1.072, 2.024] | 0.017 | 1.189 [0.846, 1.673] | 0.319 | 1.138 [0.806, 1.608] | 0.462 |
| Consistency of stool | normal | 1 [Reference] |  | 1 [Reference] |  | 1 [Reference] |  |
|  | dry stools | 1.409 [1.091, 1.818] | 0.008 | 1.211 [0.920, 1.596] | 0.172 | 1.176 [0.889, 1.556] | 0.191 |
|  | loose stools | 0.975 [0.768, 1.237] | 0.834 | 1.022 [0.792, 1.320] | 0.865 | 1.006 [0.777, 1.302] | 0.964 |
| Sensation | very smooth | 1 [Reference] |  | 1 [Reference] |  | 1 [Reference] |  |
|  | general smooth | 1.257 [1.053, 1.500] | 0.011 | 0.988 [0.815, 1.197] | 0.900 | 0.969 [0.797, 1.178] | 0.554 |
|  | difficult | 1.695 [1.331, 2.158] | <0.001 | 1.332 [1.027, 1.728] | 0.031 | 1.309 [1.003, 1.709] | 0.047 |
| **Outcome: Language impairment** | | | | | | | |
| Duration | ≤5 min | 1 [Reference] |  | 1 [Reference] |  | 1 [Reference] |  |
|  | 6-10 | 1.146 [0.961, 1.366] | 0.130 | 1.109 [0.928, 1.325] | 0.257 | 1.103 [0.922, 1.319] | 0.286 |
|  | 11-15 | 0.979 [0.714, 1.343] | 0.897 | 0.913 [0.663, 1.257] | 0.578 | 0.902 [0.653, 1.246] | 0.532 |
|  | ≥16 min | 1.628 [1.257, 2.108] | <0.001 | 1.510 [1.160, 1.966] | 0.002 | 1.485 [1.137, 1.939] | 0.004 |
| Frequency | 1/day | 1 [Reference] |  | 1 [Reference] |  | 1 [Reference] |  |
|  | >1/day | 0.989 [0.819, 1.194] | 0.907 | 1.010 [0.834, 1.223] | 0.842 | 1.003 [0.827, 1.216] | 0.975 |
|  | 1/2 days | 1.059 [0.820, 1.366] | 0.661 | 1.012 [0.780, 1.311] | 0.429 | 0.990 [0.763, 1.286] | 0.943 |
|  | 1/3 days | 1.229 [0.904, 1.672] | 0.189 | 1.170 [0.856, 1.600] | 0.325 | 1.129 [0.822, 1.550] | 0.453 |
| Consistency of stool | normal | 1 [Reference] |  | 1 [Reference] |  | 1 [Reference] |  |
|  | dry stools | 1.519 [1.193, 1.934] | 0.001 | 1.405 [1.098, 1.798] | 0.007 | 1.396 [1.088, 1.791] | 0.009 |
|  | loose stools | 1.044 [0.825, 1.322] | 0.718 | 1.027 [0.809, 1.304] | 0.824 | 1.015 [0.799, 1.290] | 0.903 |
| Sensation | very smooth | 1 [Reference] |  | 1 [Reference] |  | 1 [Reference] |  |
|  | general smooth | 1.182 [0.993, 1.408] | 0.060 | 1.102 [0.921, 1.318] | 0.290 | 1.093 [0.912, 1.309] | 0.336 |
|  | difficult | 1.395 [1.107, 1.757] | 0.005 | 1.301 [1.027, 1.649] | 0.029 | 1.264 [0.993, 1.608] | 0.057 |
| **Outcome: Executive impairment** | | | | | | | |
| Duration | ≤5 min | 1 [Reference] |  | 1 [Reference] |  | 1 [Reference] |  |
|  | 6-10 | 0.774 [0.574, 1.044] | 0.094 | 0.679 [0.492, 0.936] | 0.018 | 0.674 [0.488, 0.932] | 0.017 |
|  | 11-15 | 1.338 [0.851, 2.104] | 0.208 | 1.227 [0.745, 2.021] | 0.421 | 1.258 [0.757, 2.092] | 0.376 |
|  | ≥16 min | 1.291 [0.828, 2.012] | 0.260 | 1.101 [0.681, 1.781] | 0.695 | 1.047 [0.643, 1.705] | 0.853 |
| Frequency | 1/day | 1 [Reference] |  | 1 [Reference] |  | 1 [Reference] |  |
|  | >1/day | 1.068 [0.802, 1.422] | 0.652 | 1.156 [0.851, 1.569] | 0.354 | 1.149 [0.842, 1.566] | 0.381 |
|  | 1/2 days | 1.921 [1.303, 2.834] | 0.001 | 1.818 [1.194, 2.769] | 0.005 | 1.761 [1.151, 2.693] | 0.009 |
|  | 1/3 days | 1.460 [0.861, 2.474] | 0.160 | 1.205 [0.679, 2.138] | 0.524 | 1.119 [0.624, 2.008] | 0.706 |
| Consistency of stool | normal | 1 [Reference] |  | 1 [Reference] |  | 1 [Reference] |  |
|  | dry stools | 1.515 [0.985, 2.330] | 0.059 | 1.298 [0.811, 2.077] | 0.277 | 1.232 [0.765, 1.983] | 0.391 |
|  | loose stools | 1.325 [0.934, 1.879] | 0.115 | 1.426 [0.982, 2.071] | 0.062 | 1.368 [0.937, 1.998 | 0.104 |
| Sensation | very smooth | 1 [Reference] |  | 1 [Reference] |  | 1 [Reference] |  |
|  | general smooth | 1.604 [1.216, 2.117] | 0.001 | 1.295 [0.959, 1.747] | 0.091 | 1.270 [0.936, 1.722] | 0.125 |
|  | difficult | 2.027 [1.383, 2.971] | <0.001 | 1.502 [0.991, 2.276] | 0.055 | 1.457 [0.952, 2.230] | 0.083 |
| **Outcome: Attention impairment** | | | | | | | |
| Duration | ≤5 min | 1 [Reference] |  | 1 [Reference] |  | 1 [Reference] |  |
|  | 6-10 | 1.139 [0.930, 1.394] | 0.209 | 0.981 [0.771, 1.248] | 0.877 | 0.982 [0.769, 1.253] | 0.881 |
|  | 11-15 | 1.302 [0.926, 1.830] | 0.129 | 1.335 [0.881, 2.022] | 0.173 | 1.390 [0.909, 2.125] | 0.129 |
|  | ≥16 min | 1.811 [1.363, 2.407] | <0.001 | 1.428 [1.010, 2.020] | 0.044 | 1.408 [0.991, 2.001] | 0.056 |
| Frequency | 1/day | 1 [Reference] |  | 1 [Reference] |  | 1 [Reference] |  |
|  | >1/day | 0.831 [0.662, 1.042] | 0.109 | 1.007 [0.768, 1.321] | 0.959 | 1.031 [0.782, 1.357] | 0.831 |
|  | 1/2 days | 1.302 [0.986, 1.720] | 0.063 | 0.966 [0.692, 1.347] | 0.836 | 0.940 [0.671, 1.317] | 0.720 |
|  | 1/3 days | 1.829 [1.327, 2.522] | <0.001 | 1.189 [0.812, 1.742] | 0.373 | 1.203 [0.817, 1.772] | 0.349 |
| Consistency of stool | normal | 1 [Reference] |  | 1 [Reference] |  | 1 [Reference] |  |
|  | dry stools | 1.629 [1.250, 2.124] | <0.001 | 1.089 [0.796, 1.491] | 0.593 | 1.070 [0.779, 1.470] | 0.674 |
|  | loose stools | 1.183 [0.908, 1.542] | 0.214 | 1.326 [0.960, 1.830] | 0.087 | 1.383 [0.997, 1.917] | 0.052 |
| Sensation | very smooth | 1 [Reference] |  | 1 [Reference] |  | 1 [Reference] |  |
|  | general smooth | 1.373 [1.125, 1.676] | 0.002 | 0.840 [0.664 1.064] | 0.149 | 0.822 [0.647, 1.044] | 0.108 |
|  | difficult | 2.084 [1.627, 2.670] | <0.001 | 1.340 [0.994, 1.804] | 0.055 | 1.352 [0.996, 1.836] | 0.053 |
| Abbreviation: OR (odds ratio); CI (confidence interval) | | | | | | | |
| ^a^ Crude Model; | | | | | | | |
| ^b^ Adjusted variables: sex, age, education, residence and marital status | | | | | | | |
| ^c^ Adjusted variables: sex, age, education, residence, marital status, smoking, drinking, eating vegetables, eating fruits, physical exercise, intellectual activity, depression, hypertension, diabetes, coronary heart disease and hyperlipidemia. | | | | | | | |

| **Table S8. Mediation role of depression in the association between gut risk index and cognitive function.** | | | | | |
| --- | --- | --- | --- | --- | --- |
| Outcomes | a Estimate(95%CI) | b Estimate(95%CI) | Direct effect (c') Estimate(95%CI) | Indirect effect (a*b) Estimate(95%CI) | Proportion Mediated |
| MMSE | 0.312 (0.291, 0.405)*** | -0.086 (-0.129, -0.044)*** | -0.170 (-0.323, -0.017)* | -0.027 (-0.045, -0.013) | 13.66% |
| MoCA | 0.312 (0.219, 0.405)*** | -0.117 (-0.169, -0.065)*** | -0.288 (-0.480, -0.095)** | -0.037 (-0.059, -0.020) | 11.30% |
| Language | 0.339 (0.239, 0.439)*** | -0.188 (-0.248, -0.129)* | -0.110 (-0.349, 0.129) | -0.064 (-0.095, -0.039) | 36.64% |
| Executive | 0.377 (0.236, 0.518)*** | 0.003 (-0.001, 0.006) | -0.019 (-0.029, -0.008)*** | 0.001 (-0.001, 0.002) | / |
| *P<0.05; **P<0.01; ***P<0.001 | | | | |  |
| a: Effect of constipation on depression b: Effect of depression on outcomes c’: Direct effect of constipation on outcomes in mediation design Potential mediators explained a mediated proportion of the total effect. All models were adjusted for sex, age, education, residence, marital status, smoking, drinking, eating vegetables, eating fruits, physical exercise, intellectual activity, hypertension, diabetes, coronary heart disease and hyperlipidemia. | | | | | |

| **Table S9 The association between constipation and MCI and cognitive domains(In the covariates, age was treated as continuous variables instead of categorical variables). (n=9417)** | | | | | | |
| --- | --- | --- | --- | --- | --- | --- |
| **Characteristics** | **Model 1^a^** | | **Model 2^b^** | | **Model 3^c^** | |
|  | **OR [95% CI]** | **P value** | **OR [95% CI]** | **P value** | **OR [95% CI]** | **P value** |
| MCI | 1.450 | <0.001 | 1.193 | 0.003 | 1.159 | 0.014 |
|  | [1.304, 1.614] |  | [1.063, 1.340] |  | [1.030, 1.303] |  |
| Memory impairment | 1.275 | <0.001 | 1.145 | 0.063 | 1.123 | 0.123 |
|  | [1.116, 1.458] |  | [0.991, 1.323] |  | [0.459, 1.301] |  |
| Executive impairment | 1.514 | <0.001 | 1.394 | 0.001 | 1.371 | 0.002 |
|  | [1.260, 1.820] |  | [1.142, 1.702] |  | [1.119, 1.679] |  |
| Language impairment | 1.221 | <0.001 | 1.165 | 0.008 | 1.129 | 0.037 |
|  | [1.093, 1.364] |  | [1.041, 1.304] |  | [1.007, 1.265] |  |
| Attention impairment | 1.280 | 0.002 | 0.966 | 0.714 | 0.956 | 0.638 |
|  | [1.095, 1.498] |  | [0.803, 1.163] |  | [0.791, 1.154] |  |
| Abbreviation: OR (odds ratio); CI (confidence interval) | | | | | | |
| ^a^ Crude Model; | | | | | | |
| ^b^ Adjusted variables: sex, age, education, residence and marital status | | | | | | |
| ^c^ Adjusted variables: sex, age, education, residence, marital status, smoking, drinking, eating vegetables, eating fruits, physical exercise, intellectual activity, depression, hypertension, diabetes, coronary heart disease and hyperlipidemia. | | | | | | |

| **Table S10 Association between constipation symptoms and MCI (In the covariates, age was treated as continuous variables instead of categorical variables). (n=3344)** | | | | | | | |
| --- | --- | --- | --- | --- | --- | --- | --- |
| **Characteristics** | | **Model 1**^a^ | | **Model 2**^b^ | | **Model 3**^c^ | |
|  |  | **OR  [95% CI]** | **P value** | **OR  [95% CI]** | **P value** | **OR  [95% CI]** | **P value** |
| Duration | ≤5 min | 1 [Reference] |  | 1 [Reference] |  | 1 [Reference] |  |
|  | 6-10 | 0.988 [0.831, 1.175] | 0.894 | 0.935 [0.778, 1.123] | 0.472 | 0.931 [0.773, 1.120] | 0.447 |
|  | 11-15 | 1.137 [0.843, 1.533] | 0.400 | 1.140 [0.831, 1.566] | 0.416 | 1.141 [0.828, 1.572] | 0.420 |
|  | ≥16 min | 1.536 [1.192, 1.978] | 0.001 | 1.285 [0.980, 1.686] | 0.070 | 1.242 [0.943, 1.635] | 0.122 |
| Frequency | 1/day | 1 [Reference] |  | 1 [Reference] |  | 1 [Reference] |  |
|  | >1/day | 0.945 [0.785, 1.137] | 0.546 | 1.015 [0.835, 1.235] | 0.880 | 1.022 [0.839, 1.246] | 0.826 |
|  | 1/2 days | 1.308 [1.026, 1.667] | 0.030 | 1.111 [0.857, 1.440] | 0.426 | 1.091 [0.840, 1.416] | 0.515 |
|  | 1/>3 days | 1.746 [1.300, 2.346] | <0.001 | 1.408 [1.028, 1.930] | 0.033 | 1.377 [1.000, 1.895] | 0.050 |
| Consistency of stool | normal | 1 [Reference] |  | 1 [Reference] |  | 1 [Reference] |  |
|  | dry stools | 1.628 [1.284, 2.062] | <0.001 | 1.344 [1.043, 1.730] | 0.022 | 1.319 [1.022, 1.703] | 0.033 |
|  | loose stools | 1.208 [0.965, 1.514] | 0.100 | 1.241 [0.977, 1.576] | 0.076 | 1.230 [0.966, 1.564] | 0.096 |
| Sensation | very smooth | 1 [Reference] |  | 1 [Reference] |  | 1 [Reference] |  |
|  | general smooth | 1.180 [0.995, 1.400] | 0.057 | 0.966 [0.805, 1.159] | 0.705 | 0.949 [0.788, 1.141] | 0.576 |
|  | difficult | 1.679 [1.343, 2.098] | <0.001 | 1.317 [1.038, 1.671] | 0.024 | 1.278 [1.002, 1.630] | 0.048 |
| Abbreviation: OR (odds ratio); CI (confidence interval) | | | | | | | |
| ^a^ Crude Model; | | | | | | | |
| ^b^ Adjusted variables: sex, age, education, residence and marital status | | | | | | | |
| ^c^ Adjusted variables: sex, age, education, residence, marital status, smoking, drinking, eating vegetables, eating fruits, physical exercise, intellectual activity, depression, hypertension, diabetes, coronary heart disease and hyperlipidemia.   | **Table S11 The association between constipation and MCI and cognitive domains (further control BMI). (n=8302)** | | | | | | | | --- | --- | --- | --- | --- | --- | --- | | **Characteristics** | **Model 1^a^** | | **Model 2^b^** | | **Model 3^c^** | | | **OR [95% CI]** | **P value** | **OR [95% CI]** | **P value** | **OR [95% CI]** | **P value** | | MCI | 1.430 | <0.001 | 1.193 | 0.005 | 1.152 | 0.028 | | [1.275, 1.604] | [1.054, 1.351] | [1.016, 1.307] | | Memory impairment | 1.265 | 0.001 | 1.144 | 0.092 | 1.119 | 0.167 | | [1.095, 1.461] | [0.978, 1.337] | [0.954, 1.313] | | Executive impairment | 1.492 | <0.001 | 1.401 | 0.003 | 1.366 | 0.007 | | [1.214, 1.833] | [1.120, 1.752] | [1.088, 1.715] | | Language impairment | 1.241 | <0.001 | 1.191 | 0.004 | 1.148 | 0.026 | | [1.103, 1.396] | [1.056, 1.342] | [1.017, 1.297] | | Attention impairment | 1.309 | 0.002 | 1.019 | 0.856 | 1.001 | 0.993 | | [1.107, 1.548] | [0.836, 1.242] | [0.818, 1.225] | | Abbreviation: OR (odds ratio); CI (confidence interval) | | | | | | | | ^a^ Crude Model; | | | | | | | | ^b^ Adjusted variables: sex, age, education, residence and marital status | | | | | | | | ^c^ Adjusted variables: sex, age, education, residence, marital status, smoking, drinking, eating vegetables, eating fruits, physical exercise, intellectual activity, depression, hypertension, diabetes, coronary heart disease, hyperlipidemia and BMI. | | | | | | | | | | | | | | |

| **Table S12. Association between constipation symptoms and MCI (further control BMI). (n=3344)** | | | | | | |  |
| --- | --- | --- | --- | --- | --- | --- | --- |
| **Characteristics** | | **Model 1^a^** | | **Model 2^b^** | | **Model 3^c^** | |
|  |  | **OR  [95% CI]** | **P value** | **OR  [95% CI]** | **P value** | **OR  [95% CI]** | **P value** |
| Duration | ≤5 min | 1 [Reference] |  | 1 [Reference] |  | 1 [Reference] |  |
|  | 6-10 | 0.982 [0.822, 1.173] | 0.840 | 0.924 [0.765, 1.117] | 0.414 | 0.918 [0.758, 1.111] | 0.379 |
|  | 11-15 | 1.132 [0.830, 1.543] | 0.433 | 1.149 [0.825, 1.591] | 0.419 | 1.147 [0.823, 1.600] | 0.418 |
|  | ≥16 min | 1.516 [1.167, 1.968] | 0.002 | 1.259 [0.952, 1.666] | 0.106 | 1.223 [0.921, 1.623] | 0.164 |
| Frequency | 1/day | 1 [Reference] |  | 1 [Reference] |  | 1 [Reference] |  |
|  | >1/day | 0.952 [0.787, 1.153] | 0.617 | 1.034 [0.845, 1.265] | 0.749 | 1.045 [0.852, 1.281] | 0.676 |
|  | 1/2 days | 1.300 [1.012, 1.670] | 0.040 | 1.103 [0.844, 1.441] | 0.474 | 1.078 [0.823, 1.412] | 0.584 |
|  | 1/>3 days | 1.667 [1.230, 2.259] | 0.001 | 1.381 [0.999, 1.909] | 0.051 | 1.356 [0.976, 1.883] | 0.069 |
| Consistency of stool | normal | 1 [Reference] |  | 1 [Reference] |  | 1 [Reference] |  |
|  | dry stools | 1.617 [1.268, 2.062] | <0.001 | 1.351 [1.042, 1.751] | 0.023 | 1.328 [1.022, 1.726] | 0.034 |
|  | loose stools | 1.243 [0.986, 1.568] | 0.066 | 1.272 [0.995, 1.626] | 0.055 | 1.261 [0.984, 1.616] | 0.067 |
| Sensation | very smooth | 1 [Reference] |  | 1 [Reference] |  | 1 [Reference] |  |
|  | general smooth | 1.192 [1.000, 1.420] | 0.050 | 0.962 [0.797, 1.161] | 0.688 | 0.944 [0.780, 1.142] | 0.550 |
|  | difficult | 1.647 [1.309, 2.073] | <0.001 | 1.293 [1.012, 1.652] | 0.040 | 1.257 [0.979, 1.614] | 0.073 |
| Abbreviation: OR (odds ratio); CI (confidence interval) | | | | | | | |
| ^a^ Crude Model; | | | | | | | |
| ^b^ Adjusted variables: sex, age, education, residence and marital status | | | | | | | |
| ^c^ Adjusted variables: sex, age, education, residence, marital status, smoking, drinking, eating vegetables, eating fruits, physical exercise, intellectual activity, depression, hypertension, diabetes, coronary heart disease, hyperlipidemia and BMI. | | | | | | | |
